# Effect of metabolic syndrome and its components on survival in colorectal cancer: a prospective study

**DOI:** 10.12861/jrip.2015.05

**Published:** 2015-03-01

**Authors:** Ali Ahmadi, Mehdi Noroozi, Mohamad Amin Pourhoseingholi, Seyyed-Saeed Hashemi-Nazari

**Affiliations:** ^1^Department of Epidemiology and Biostatistics, School of Health, Shahrekord University of Medical Sciences, Shahrekord, Iran; ^2^Department of Epidemiology, School of Health, Shahid Beheshti University of Medical Sciences, Tehran, Iran; ^3^Gastroenterology and Liver Disease Research Center, Shahid Beheshti University of Medical Sciences, Tehran, Iran; ^4^Department of Epidemiology, School of Public Health, Shahid Beheshti University of Medical Sciences, Tehran, Iran

**Keywords:** Metabolic syndrome, Colorectal cancer, Cox proportional model

## Abstract

**Introduction:** Metabolic syndrome (MetS) may affect prognosis of the patients diagnosed with colorectal cancer (CRC).

**Objectives:** This study was aimed to design a model and to examine the prognostic effect of MetS on survival time in the patients with CRC.

**Patients and Methods:** Data were collected from 1127 cases of CRC from Cancer Registry Center of the Research Institute of Gastroenterology and Liver Disease, Shahid Beheshti University of Medical Sciences, Tehran, Iran. In this cohort study, patients were divided into two groups based on the presence of MetS. We tested the prognostic value of MetS in the patients by Cox proportional hazard modeling.

**Results:** Mean ± standard deviation of the patients’ age at diagnosis in MetS group and non-MetS group was 56 ± 13 years old and 53 ± 15 years old respectively. Tumor stage as an independent variable affected CRC survival. The mean survival time of the MetS and non-MetS groups was 23 and 27 months respectively. Independent variables like tumor stage (hazard ratio [HR], 1.76; 95% CI, 0.29–0.90) and educational level (HR, 0.50; 95% CI, 0.23–0.97) had significant effect on CRC survival and MetS (HR, 0.95; 95% CI, 0.52–1.5), tumor size (HR, 1.390; 95% CI, 1.237–1.560), family history, age, gender, and smoking had non-significant effect on CRC survival.

**Conclusion:** MetS could be a prognostic factor for survival in the patients with CRC. The results suggested that effect of MetS was not significant.

***Implication for health policy/practice/research/medical education*:**

Although metabolic syndrome (MetS) has been investigated as a potential risk factor for colorectal cancer (CRC) and could be a prognostic factor for survival in the patients with CRC, but its effect was not significant and hence the way could be paved for decision makers and planners in health system.

## Introduction

Metabolic syndrome (MetS) may increase the risk of colorectal cancer (CRC) through different biological mechanisms particularly those that are associated with insulin resistance. The underlying mechanism is still unknown but hyperinsulinemia could contribute to enhancing free or bio-available concentrations of insulin (1,2). MetS comprises a variety of risk factors for type 2 diabetes and cardiovascular diseases, including obesity, impaired glucose, hypertension, hypertriglyceridemia, and decrease in high density lipoprotein and cholesterol levels. Central elements that are associated with MetS and obesity, such as hyperinsulinemia and insulin resistance, contribute importantly to the process of getting cancerous in breast cancer, prostate cancer, endometrial cancer, and CRC (3-5). A meta-analysis showed that MetS was associated with the increase in the risk of CRC-related mortality (2,5). Each year, one million new cases of CRC are diagnosed and 500 000 deaths occur due to its worldwide. In the United States CRC is the third leading cancer in both genders and the third cancer resulting in death, comprising 9% of the whole mortalities from cancers (6). According to the cancer registry, CRC incidence in five continents varies from 3% in Africa up to 40% in the United States. In Europe, CRC incidence varies from 12.1% in Belarus to 30.5% in Italy (7,8). In addition, CRC was the most frequently diagnosed cancer and the second common cause of the death from cancers and comprised 13% of all new cancer cases in both genders in organization for economic cooperation and development member countries in 2008. MetS, alongside cardiovascular risks, has been offered in some epidemiological studies as being associated with several cancers including CRC. There is an overlap among risk factors for MetS, diabetes mellitus, and CRC (9,10).

In Iran according to the national cancer registry’s report in 2009, acoustic startle response (ASR) of CRC in women was 10.89 (ranked as the third) and in men was 11.31 (ranked as the fifth). In a study carried out by Ansari *et al*. in 2005, in the provinces of Ardabil, Gilan, Mazandaran, Golestan, and Kerman, ASR of CRC was 8.2 in men and 7 in women, which is approximate to the national cancer registry’s report (11-13).

Although MetS has been investigated as a potential risk factor for CRC (13), various studies have obtained conflicting results relevant to MetS-associated CRC risk of death. However, a study in China showed that MetS had no obvious effect on observable survival and recurrence free rate (9,14,15). Several meta-analysis have been conducted about association between MetS and CRC and reported heterogeneity and homogeneity (2,5,16,17). To the best of our knowledge, no study of CRC survival and MetS to date has been conducted in Iran (18-20).

## Objectives

The aim of this study is to examine whether the MetS is associated with survival of the patients with CRC.

## Patients and Methods

The present study is a prospective, cohort one. Data were obtained from cancer registry center of the research institute of gastroenterology and liver disease, Shahid Beheshti University of Medical Sciences, Tehran, Iran. The patients from public and private collaborative hospitals were treated and referred to the cancer registry. This study is based on the data of cancer registry including demographic factors (sex, age, education, job, etc.), medical records, family history, and diagnosis information (symptoms at diagnosis, tumor metastasis, grade of tumor, etc.). All patients with CRC diagnosis by the pathology report of cancer registry were considered as eligible. Finally, 1219 patients (802 [66%] with colon cancer, 392 [32%] with rectal cancer, and 25 [2%] with unknown cause) were enrolled. The follow-up duration was determined from the date of diagnosis up to first of October 2008 as the time of the death from the disease (as the exact failure time) or survival (as the censoring time). First of January 2002 was considered as the start time of the study. Deaths were confirmed through calling relatives of the patients by telephone. On few (2%) CRC patients, no clear information was obtained about the cause of death except the date of death; therefore, their data were excluded from the analysis. There were 1127 patients (including 690 men and 437 women) with complete follow-up data. These patients were divided into two groups based on the presence of MetS. A physician interviewed the participants separately, performed a physical examination, and obtained a detailed medical history. The data were recorded carefully. Body weight (kg) and height (m) were measured to compute body mass index (BMI). Blood pressure was measured using the right arm and a standard mercury sphygmomanometer in sitting position after a 5-minute rest, and the mean systolic and diastolic blood pressure values of the two measurements were recorded. After measurement of blood pressure, while the participant was fasting, a venous blood sample was taken for determination of the serum glucose. For all patients and based on medical file, the data on age, sex, smoking, size of the tumor, histological type, degree of differentiation (low degree as the referent) or tumor, node, metastasis (TNM) stage, and alcohol drinking history, separately or in combination, were used in the analysis.

### Ethical issues

(1) The research followed the tenets of the Declaration of Helsinki; (2) informed consent was obtained; (3) the research was approved by ethical committee of Shahid Beheshti University of Medical Sciences. This study with approval No. 686 issued by Shahid Beheshti University of Medical Sciences Ethics Committee was conducted after obtaining the informed consent from the patients.

### Statistical analysis

Continuous variables in this study were expressed as mean ± standard deviation (SD). The variables that were statistically significant by univariate analysis were included in a multivariate analysis, confirmed by Cox regression (Cox proportional hazards model) with forward stepwise selection of covariates and with enter and remove limits of P values of less than 0.050 and greater than 0.100, respectively. Cox proportional hazards model was determined as primary mode of analysis, through which adjustment for age, smoking, and alcohol drinking and other variables was performed. The assumption of proportionality of the hazards was tested and found as being not violated. In the final model, P values of less than 0.05 were considered statistically significant. Stata 12 software was used for the statistical analysis.

## Results

The mean (± SD) follow-up time for the patients with CRC was 26 (± 25) months. The mean (± SD) age at diagnosis was 54 (± 14) years in CRC patients. Mean (± SD) age at diagnosis was 56 (± 13) years in MetS group and was 53 (± 15) years in non-MetS. There were non-significant differences in the age at diagnosis between the two groups (P > 0.060). In the MetS group, 187 tumors were highly differentiated, 52 were moderately differentiated, and 13 were slightly differentiated. In the non-MetS group, 273 tumors were highly differentiated, 177 were moderately differentiated, and 46 were slightly differentiated; no significant difference in degree of differentiation was observed between the two groups (Chi-square, 0.502; P = 0 .778). Mean (± SD) size of tumor was 76 (± 9) and 75 (±13) mm3 in MetS and non-MetS groups, respectively. The mean survival time in the MetS and non-MetS groups was 23 and 27 months respectively. Survival time was shorter in the MetS compared to the non-MetS group. Survival curve revealed that survival of the MetS group was worse than the non-MetS group. Variables examined by univariate analysis for overall survival of CRC included sex, age, smoking, size of the tumor, histological type, degree of differentiation (low degree as the referent) or TNM stage, with MetS (or non-MetS group as the referent), family history, education level, alcohol drinking history, and marital status. Then variables obtained statistically significant by univariate analysis (P < 0.05) were included in a multivariate analysis. Independent variables like tumor stage (hazard ratio [HR], 1.76; 95% CI, 0.29–0.90) and educational level (HR, 0.50; 95% CI, 0.23–0.97) had significant effect on CRC survival and MetS (HR, 0.95; 95% CI, 0.52–1.5), tumor size (HR, 1.390; 95% CI, 1.237– 1.560), family history, age, gender, and smoking had non-significant effect on CRC survival ((Table 1).

**Table 1:** Univar iate and multivariate Cox model for the effect MetS and its components on survival in colorectal cancer

**Characteristics**		**Hazard Ratio**		**P value**		**95% CI**	
		**Crude**	**Adjusted**	**Univariate**	**Multivariate**	**Univariate**	**Multivariate**
MetS	Yes	0.81	0.95	0.05	0.6	0.06-3.78	0.52-1.22
	No	1	1				
SEX	Male	0.86	1.09	0.3	0.7	0.66-1.4	0.67-1.77
	Female	1	1
Marital status	Married	1	1	-	-	-	0.88-3.49
	Single	1.92	1.75	0.004	0.1	1.23-3.0	
Education	Illiterate	1	1	1
	Primary school	0.68	0.5	0.05	0.03	0.465-1.0	0.26-.94
	High school	0.68	0.4	0.07	0.02	0.45-1.05	0.20-0.89
	university	0.67	0.6	0.08	0.12	0.43-1.04	0.36-1.2
Tumor grade	grade II	1.46	1.2	0.03	0.4	1.03-2.08	0.73-1.9
	III to IV	1.89	2.2	0.01	0.01	1.14-3.33	1.9-4.4
Age at diagnosis		1.03	1.00	0.3	0.9	0.9-1.03	0.98-1.01
Hypertension		0.82	0.83	0.4	0.6	0.5-1.64	0.42-1.64
Diabetes		0.9	1.45	0.7	0.3	0.5-1.64	0.71-2.95
High body mass index		0.71	0.62	0.001	0.006	0.35-6.8	0.44-8.7

## Discussion

Cancer is the second leading cause of death in the world and is the third leading cause of mortality in Iran. Regarding this purpose, we conducted this study on 1227 Iranian CRC patients to evaluate the effect of MetS on survival of CRC. The incidence of MetS is increasing due to urbanization, aging, diet structure, and lifestyle (21-24). Recent studies show a relationship between diet structure and the prevalence of CRC (25,26). However, the studies about the association between CRC prognosis and MetS have been less conducted. Marrero *et al*. reported a moderate association of post load plasma glucose and insulin resistance syndrome with CRC and found that the survival rate of breast cancer in the MetS group was higher than that of the control group; however, there are few reports on the association between MetS and CRC. In our study, no obvious differences were observed in tumor differentiation between the two groups. Moreover, our study showed that the mean survival time of the MetS group was shorter than that of the non-MetS group, with a statistically non-significant difference. However, results of some other studies showed the mean survival time of the MetS group was shorter than that of the non-MetS group, with a statistically significant difference (16). This difference might be due to the definition of MetS or mode of survival estimation (i.e. diagnosis to death or treatment to death). Shen *et al*. found that MetS and survival of colon cancer were related but MetS had no effect on rectal cancer (10). Also, Colangelo *et al*. reported no association between MetS and prognosis of CRC (27,28). The difference in the findings could be due to different definitions of MetS and/ or the method of survival estimation (from the diagnosis till death or from treatment till death). Furthermore, in our multivariate analysis of survival, we found that worse TNM stage and size of the tumor were important independent risk factors for survival in CRC, which is consistent with others’ findings.

## Limitations

There were some limitations in our study. The information gathered about opoid and drug abuse history and smoking was incomplete, based on only two categories of “*never*” and “*current or past user*”, and qualitative; quantitative data about these two factors could yield more exact results. There was no data of dietary habit of the patients, as well.

## Conclusion

Our study showed that the mean survival time of the MetS group was shorter than that of the non-MetS group, with a statistically non-significant difference. MetS could be a prognostic factor for survival in the patients with CRC. The results suggested that effect of MetS was not significant.

## Acknowledgments

Hereby we greatly appreciate the valuable contribution of Shahid Beheshti University of Medical Sciences and Cancer Registry Center of Research Institute of Gastroenterology and Liver Disease to this study.

## Authors’ contributions

All authors contributed to design of the research. AA, MN, MP and SHN conducted the research. AA and SHN analyzed the data. AA, MN and SHN prepared the manuscript. All authors read, revised, and approved the final manuscript.

## Conflict of interests

The authors declare no conflict of interests.

## Ethical considerations

Ethical issues (including plagiarism, misconduct, data fabrication, falsification, double publication or submission, redundancy) have been completely observed by the authors.

## Funding/Support

This study with approval No. 686 issued by Shahid Beheshti University of Medical Sciences. Data collection for this research was supported by the Cancer Registry Database of the Research Center for Gastroenterology and Liver Diseases affiliated to Shahid Beheshti University of Medical Sciences. The funding sources played no role in the study design, data analysis, and manuscript writing, or in the decision to submit this manuscript for publication.
